# Calcifying fibrous tumour—a rare cause of anaemia

**DOI:** 10.1093/jscr/rjaa573

**Published:** 2021-01-18

**Authors:** Nurul Nadhirah Abd Kahar, Ahmed Al-Mukhtar

**Affiliations:** General Surgery Department, Sheffield Teaching Hospitals NHS Foundation Trust, Northern General Hospital, Sheffield, UK; General Surgery Department, Sheffield Teaching Hospitals NHS Foundation Trust, Northern General Hospital, Sheffield, UK

## Abstract

Calcifying fibrous tumour (CFT) is a rare benign tumour with non-specific anatomical distribution. We describe a case of a patient who presented with chronic generalised fatigue secondary to anaemia. Her symptoms did not improve while being on oral iron replacement therapy. Further endoscopic investigations were unremarkable. She had a computed tomography scan showing masses in the right pleural base and in the spleen. She then underwent splenic biopsy that only showed inflammatory changes. As her symptoms persisted, she was worked up for elective laparoscopic splenectomy during which she was found to have multiple peritoneal deposits. Biopsies were taken and the splenectomy was abandoned. The biopsies eventually showed changes consistent with CFT. This was conclusive for diagnosis of multifocal CFT.

## INTRODUCTION

Calcifying fibrous tumour (CFT) was initially described in childhood cases. Its aetiology remains unclear, and few postulations have been made. The symptoms are often non-specific. Owing to its potential to be multifocal, the diagnosis can be clinically challenging. However, CFT has a specific histological finding that becomes the gold standard to confirm the diagnosis. Further studies are needed be to clarify the pathophysiology of this rare disease.

## CASE REPORT

A 57-year-old female was referred from primary care with a 3-year history of generalised fatigue. She has background of ichthyosis, lichen sclerosis, asthma, depression and chronic herpes simplex virus infection. She had hysterectomy due to abnormal smear cells, which was then proven benign on histology. She was initially found to have microcytic anaemia with low ferritin level which did not improve despite oral iron replacement. Both oesophago-gastro-duodenoscopy and colonoscopy were unremarkable. A subsequent computed tomography (CT) scan of the abdomen and pelvis showed a splenic mass with a central area of calcification and a calcified 35 mm pleural-based nodule at the right costophrenic angle.

**Figure 1:**
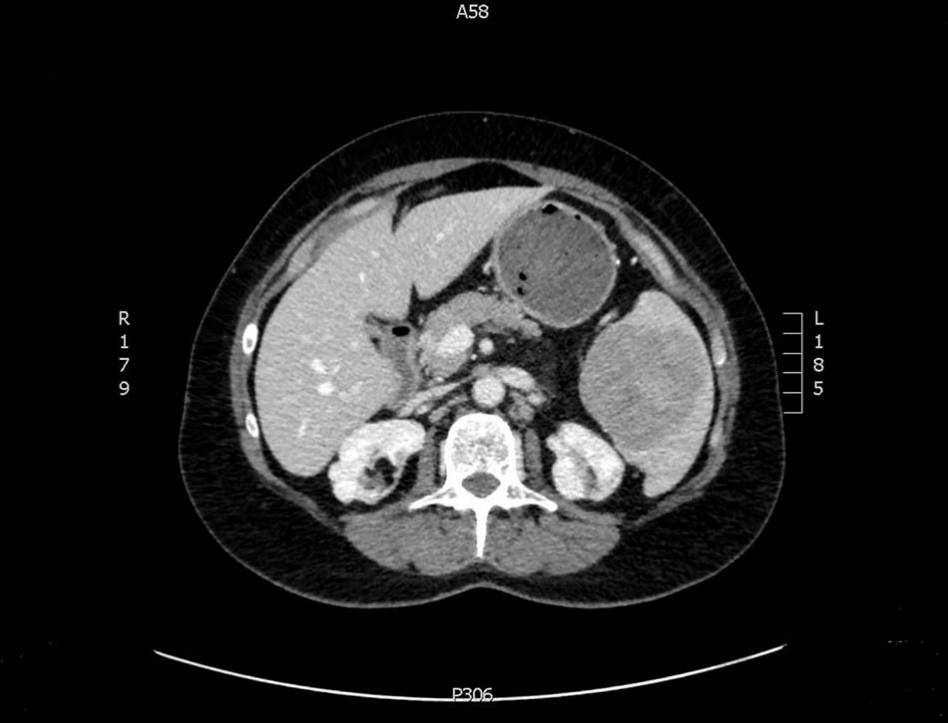
CT image of a splenic mass

**Figure 2:**
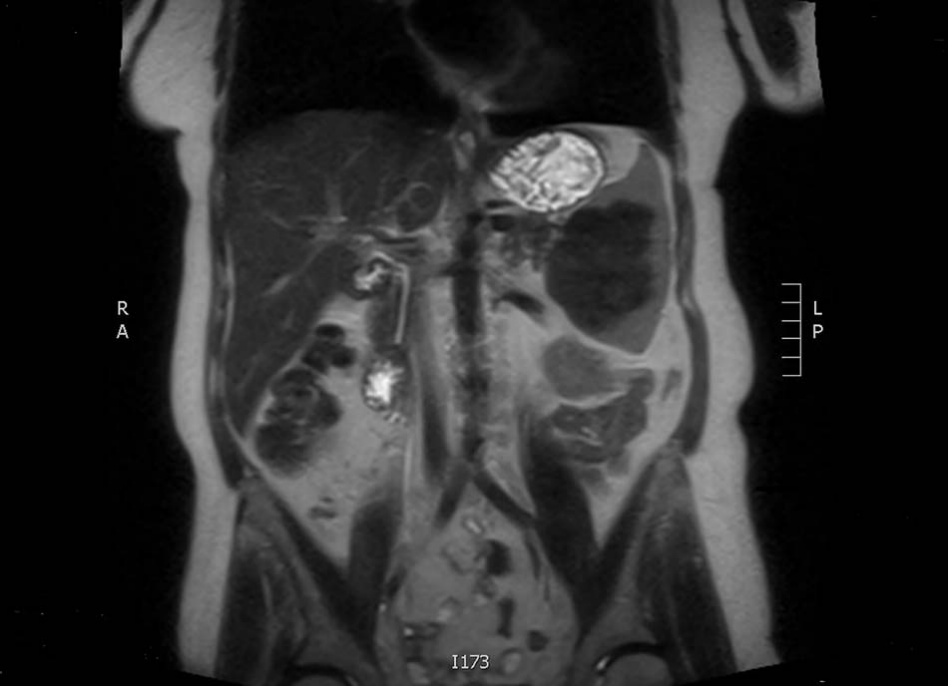
MRI image of a splenic mass

The latter was discussed in the lung multi-disciplinary team (MDT) meeting. They concluded that the pleural mass was most likely due to post-inflammatory changes. In absence of concerning features, further monitoring or intervention was deemed unnecessary. The splenic mass was then discussed in the hepatobiliary MDT meeting. They recommended obtaining tissue biopsy to aid diagnosis. However, it only showed fibrotic tissue with possible granulomas. The indeterminate histology prompted a repeat CT scan and further magnetic resonance imaging (MRI) to assess the mass further and exclude the possibility of lymphoma. These scans showed stable appearance of the splenic lesion at 6 cm (Figs 1 and 2). The CT scan also picked up numerous small calcifications scattered throughout the peritoneal cavity.

**Figure 3:**
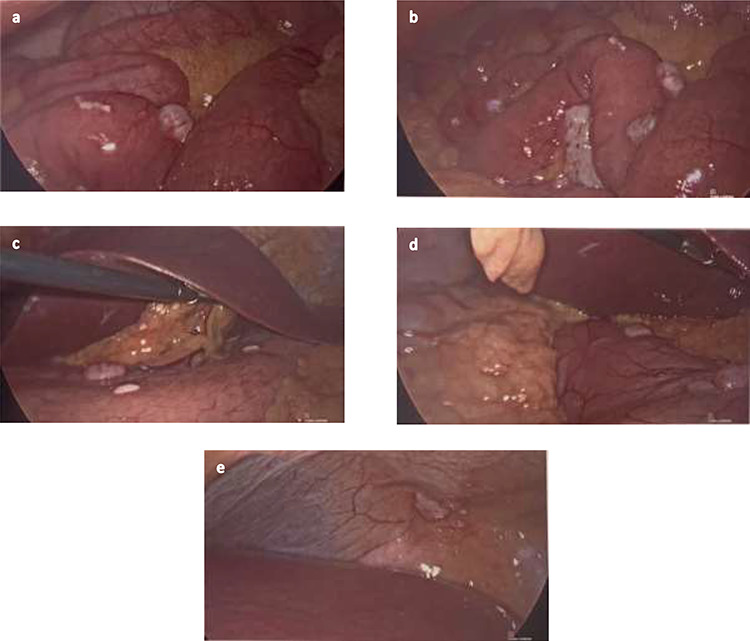
(**a** and **b**) Nodules found on small bowel mesentery. (**c** and **d**) Nodules on the stomach. (e) Nodule on the peritoneal cavity.

She then had a diagnostic laparoscopy with a view of performing a splenectomy. However, widespread nodules were found in the peritoneum and throughout the abdomen as shown in Fig. 3. There was no evidence of malignancy found on laparoscopy. Given these findings, further biopsies of the nodules were obtained and splenectomy was abandoned.

**Figure 4:**
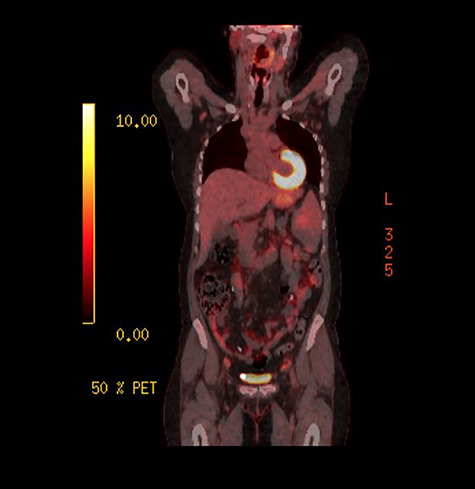
PET scan showing low-grade activity in the spleen

As the investigations thus far were non-conclusive, positron emission tomography (PET) scan was performed while awaiting for the result of the peritoneal biopsies. This was performed to look for possible primary malignancy. The splenic lesion demonstrated low-grade activity, below that typically seen in high-grade haematological malignancy as seen on Fig. 4. Combined with calcifications throughout the peritoneal cavity, an inflammatory or infective cause would be more likely.

The peritoneal nodules biopsies were reported as well-marginated with prominent areas of hyalinization and numerous foci of psammomatous calcifications. This is consistent with the diagnosis of CFT. With widespread CFT lesions, the patient would not benefit from splenectomy. She is being followed up with yearly CT scan to monitor the lesions and to continue with iron supplementation.

## DISCUSSION

CFT was initially reported by Rosenthal and Abdul-Karim in 1988 as ‘childhood tumour with psammoma bodies’ in two childhood cases [1]. Fetsch *et al*. [2] then described 10 similar cases in individuals with age range of 1–33 years, and the term ‘calcifying fibrous pseudotumour’ was introduced. The term ‘pseudotumour’ was used at that time as the underlying process was most likely inflammatory [2]. World Health Organisation recognized its potential for local recurrence [3] and renamed it as ‘calcifying fibrous tumour’ [4].

CFT can occur as solitary or multiple lesions. A review by Chorti *et al*. [5] looked into 161 cases reported based on the anatomical location. The most common ones include the stomach, small intestine, pleura, oesophagus, neck, mesentery, mediastinum and peritoneum. Due to this wide distribution, patients present with non-specific symptoms such as anorexia, weight loss, generalised fatigue, dyspnoea and progressive weakness. However, individuals can also present with specific symptoms related to organs involved [5]. In our case, the patient initial presenting complaint was generalised fatigue and she was found to have anaemia. Subsequent imaging revealed pleural mass and splenic mass. Interestingly she did not complaint of any breathing problem or abdominal discomfort despite the above findings.

The cause and pathogenesis of CFT is unclear but some hypotheses have been suggested in the past. Chen reported a case of two sisters with multifocal peritoneal CFT suggesting that there might be genetic susceptibility for CFT, although common environmental factors cannot be discounted [6]. In 1999, Van Dorpe *et al.* [7] reported that CFT can represent a late stage of inflammatory myofibroblastic tumour (IMT) as the histology showing features of both. Histology of IMT lesions is commonly more cellular, less hyalinised and lack in calcification [8].

CFT has also been reported to be associated with sclerosing angiomatoid nodular transformation (SANT) of the spleen [9, 10]. This is a rare benign lesion of the spleen with unknown aetiology. The nodules of SANT are thought to show abnormal reactionary transformation of red pulp triggered by an exaggeration of stromal response, making it more likely to be reactive rather than neoplastic [9]. A splenectomy would be required for the definitive diagnosis of SANT as a core biopsy will be inadequate.

Ideally, local CFT can be resected when diagnosed [5, 8] but in cases of multifocal CFT this might prove a challenge. Some argued that further resection of other masses will not be beneficial if the patients are asymptomatic [8]. Generally, CFT has good prognosis due to its benign nature. However, there has been documentation of a few cases of patients with recurrence during the follow-up period [2, 5]. There has been no mortality reported due to CFT [5].

## CONCLUSION

CFT is a rare benign tumour with non-specific anatomical distribution. Due to this, patients tend to present with non-specific symptoms and the tumour is usually picked up on radiological imaging. Histology is the gold standard to diagnose CFT and this can be confirmed by experienced pathologists. The aetiology of CFT is still unknown and further studies should be implemented to identify the pathophysiology of the disease.

## References

[1] Rosenthal NS, Abdul-Karim FW Childhood fibrous tumor with psammoma bodies. Clinicopathologic features in two cases. Arch Pathol Lab Med 1988;112:798–800. 3395217

[2] Fetsch JF, Montgomery EA, Meis JM Calcifying fibrous pseudotumor. Am J Surg Pathol 1993;17:502–8. 8470765 10.1097/00000478-199305000-00010

[3] Nascimento AF, Ruiz R, Hornick JL, Fletcher CDM Calcifying fibrous ‘pseudotumor’: Clinicopathologic study of 15 cases and analysis of its relationship to inflammatory myofibroblastic tumor. Int J Surg Pathol 2002;10:189–96. 12232572 10.1177/106689690201000304

[4] Fletcher F, Bridge CDM, Hogendoorn JA, Mertens P WHO classification of tumours of soft tissue In: WHO Classification of Tumours of Soft Tissue and Bone, Vol. 46, 4th edn. Boston, MA, US, 2013, 10–2.

[5] Chorti A, Papavramidis TS, Michalopoulos A Calcifying fibrous tumor. Med (United States) 2016;95:e3690. 10.1097/MD.0000000000003690 PMC4902420 27196478

[6] Chen KTKMD Familial peritoneal multifocal calcifying fibrous tumor. Am J Clin Pathol 2003;119:811–5. 12817428 10.1309/MXC6-TWEL-UUH4-20W0

[7] Van Dorpe J, Ectors N, Geboes K, D’Hoore A, Sciot R Is calcifying fibrous pseudotumor a late sclerosing stage of inflammatory myofibroblastic tumor? Am J Surg Pathol 1999;23:329–35. 10078925 10.1097/00000478-199903000-00013

[8] Azam F, Chatterjee M, Kelly S, Pinto M, Aurangabadkar A, Latif M, et al. Multifocal calcifying fibrous tumor at six sites in one patient: a case report. World J Surg Oncol 2014;12:235. 25070647 10.1186/1477-7819-12-235 PMC4127171

[9] Kuo TT, Chen TC, Lee LY Sclerosing angiomatoid nodular transformation of the spleen (SANT): clinicopathological study of 10 cases with or without abdominal disseminated calcifying fibrous tumors, and the presence of a significant number of IgG4+ plasma cells. Pathol Int 2009;59:844–50. 20021608 10.1111/j.1440-1827.2009.02456.x

[10] Lee JC, Lien HC, Hsiao CH Coexisting sclerosing angiomatoid nodular transformation of the spleen with multiple calcifying fibrous pseudotumors in a patient. J Formos Med Assoc 2007;106:234–9. 17389168 10.1016/S0929-6646(09)60245-X

